# *Athyrium bipinnatum* K.Hori (Athyriaceae), a new cornopteroid fern from Japan

**DOI:** 10.3897/phytokeys.148.51589

**Published:** 2020-05-26

**Authors:** Kiyotaka Hori

**Affiliations:** 1 The Kochi Prefectural Makino Botanical Garden, Kochi, Japan Kochi Prefectural Makino Botanical Garden Kochi Japan

**Keywords:** *
Athyrium
*, *
Cornopoteris
*, Japan, new species

## Abstract

I describe *Athyrium bipinnatum***sp. nov.** and discuss morphological differences between closely related species. The new species is endemic to Japan, occurring on the islands of Honshu, Kyushu, and Shikoku. Based on the criteria of the International union for conservation of nature and natural resources, this new species is here considered endangered.

## Introduction

*Cornopteris* Nakai is a small Asian (Himalayas, East and Southeast Asia) genus of terrestrial ferns that used to be recognized by many pteridologists (e.g., Nakai 1930; Ito 1939; Ching 1945; Holttum 1958; Tagawa 1959; Kato 1977, 1979), on the basis of fleshy stipes, corniculate leaf axes, and exindusiate sori.

Regarding its phylogenetic relationships, Ching (1945) regarded it to be “a little offshoot of the exindusiate *Diplazium*.” Based on the presence of J-shaped sori in some species of *Cornopteris*, as well as on stipe features and spinulose midribs of the laminae, Kato (1977) concluded that *Cornopteris* was actually more closely related to *Athyrium* than to *Diplazium*. Serizawa (1981) agreed with Kato’s conclusion and subsumed *Cornopteris* in *Athyrium*. To corroborate his taxonomic decision of lumping the two genera, Serizawa (1981) also highlighted the existence of natural hybrids between them. These are: Athyrium × cornopteroides Sa.Kurata (*Cornopteris opaca* (D.Don) Tagawa × *Athyrium kuratae* Seriz.), Athyrium × glabrescens Seriz. (*Cornopteris decurrentialata* (Hooker) Nakai × *A. kuratae*), and Athyrium × petiolatum Sa.Kurata (*C. opaca* × *Athyrium yakusimense* Tagawa). Sano et al. (2000) showed monophyly of the genus *Cornopteris* based on *rbcL* gene phylogeny, but Adjie et al. (2008) showed *A. distentifolium* placed in the same clade of *Cornopteris* in *rbcL* phylogeny. Therefore, *Cornopteris* spp. have been classified in *Athyrium* (Athyriaceae) based on DNA phylogenies (Rothfels et al. 2012, PPG I 2016, Ebihara 2017). However, Moran et al. (2019) distinguished the genus *Cornopteris* from *Athyrium* based on plastid DNA phylogeny. In the present study, *Cornopteris* spp. are treated as members of *Athyrium*.

Cornopteroid species of the genus *Athyrium* are mainly distributed in the Himalayas, in East and Southeast Asia. The exact number of species is unknown. Kato (1979) recognized nine species of cornopteroid ferns, whereas Zhaorong and Kato (2013) recognized 16 species only in China.

**Figure 1.**
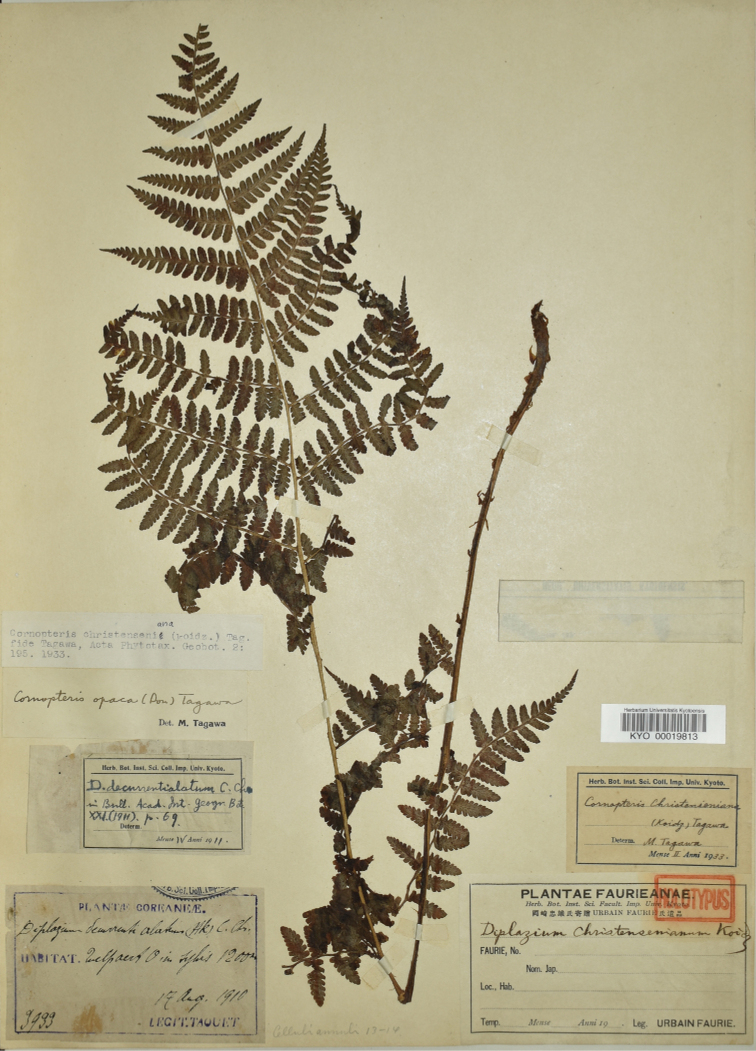
Holotype of Diplazium × christensenianum Koidz.

The following is a brief history of the classification of one *Athyrium* species, namely Athyrium × christensenianum (Koidz.) Seriz., which is partly the focus of the present study. Athyrium × christensenianum was first described by Koidzumi (1924) as *Diplazium christensenianum* Koidz., from Jeju (Quelpaert)-Island, South Korea (Fig. 1). Kato (1979) synonymized *Cornopteris hakonensis* Nakai, from Hakone, Japan (Nakai 1930, Fig. 2) under *Cornopteris christensenianum*. Subsequently, Serizawa (1981) transferred it to *Athyrium* and created the nothospecific name A. × christensenianum. The hybrid origin of this species has been pointed out by many authors (Kurita 1964, Hirabayashi 1970, Kato 1979, Park and Kato 2003). According to these authors (Kurita 1964, Hirabayashi 1970, Kato 1979, Park and Kato 2003), this is a triploid “species” that has resulted from a cross between diploid sexual *Athyrium crenulatoserrulatum* Makino and tetraploid sexual *Athyrium decurrentialatum* (Hook.) Copel. It remains unclear whether A. × christensenianum is merely a sterile hybrid or an independent fertile species. Kato (1979) categorized it as being of a “hybrid origin species” between *A. crenulatoserrulatum* Makino and *A. decurrentialatum* because it has intermediate morphological characteristics between these two species such as serrated segments, subcartilaginous margins of the blade, and elliptical sori. On the other hand, Serizawa (1981) treated this “species” as a sterile “hybrid.” The present study agrees with the treatment of A. × christensenianum by Serizawa (1981).

**Figure 2.**
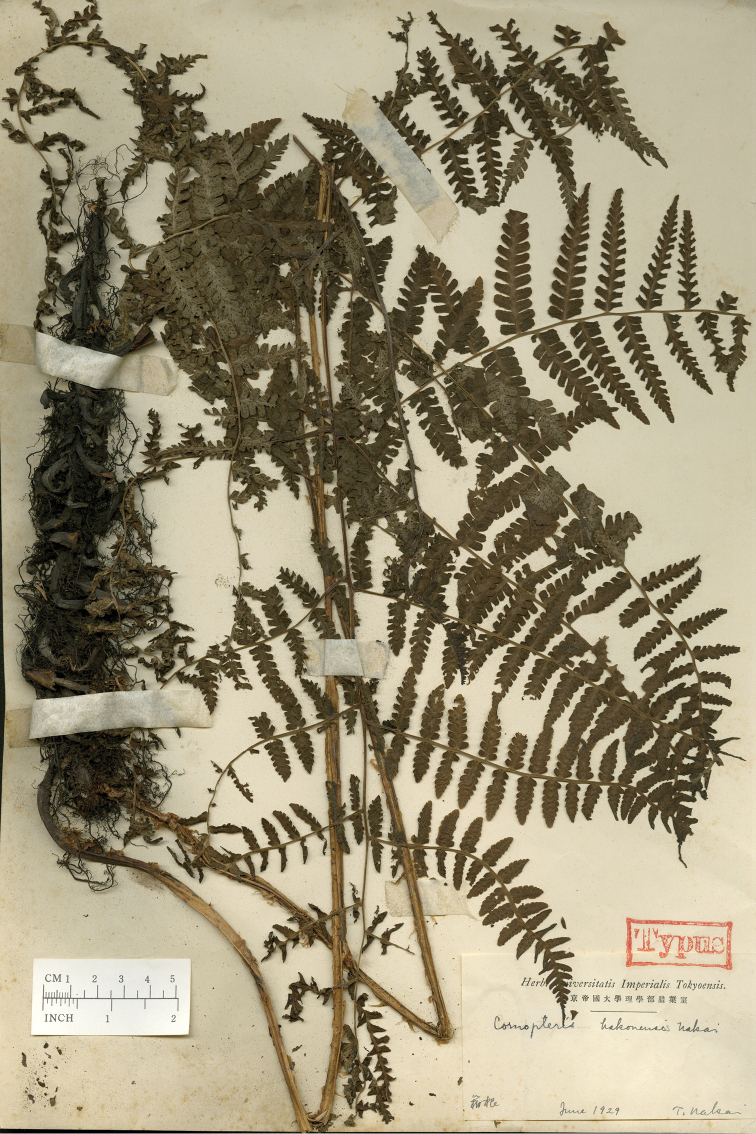
Holotype of *Cornopteris hakonensis* Nakai.

**Figure 3.**
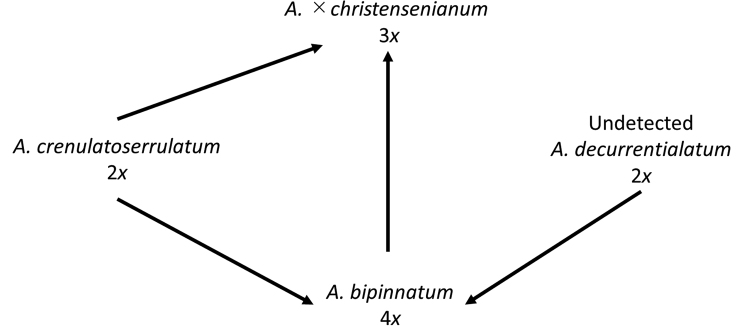
Relationships of *Athyrium bipinnatum* and its related species.

Recent studies have reported complicated relationships in the A. × christensenianum complex. First, Hori and Murakami (2019) reported a tetraploid sexual cytotype of A. × christensenianum (as the independent species *A. christensenianum*). Subsequently, Hori (2019) reported that tetraploid sexual A. × christensenianum had one allele of *A. crenulatoserrulatum* and one of *A. decurrentialatum*, each in the biparental inherited nuclear DNA marker of the gene *AK1*. He also found that triploid A. × christensenianum had two alleles of *A. crenulatoserrulatum* and one allele of *A. decurrentialatum*. Therefore, he suggested two hypotheses: (1) tetraploid sexual A. × christensenianum originated from the hybridization of diploid sexual *A. crenulatoserrulatum* with an ancestral or extinct diploid *A. decurrentialatum*; and (2) triploid A. × christensenianum originated from the hybridization of diploid sexual *A. crenulatoserrulatum* and tetraploid sexual A. × christensenianum (Fig. 3). In the present study, the unclear taxonomy of A. × christensenianum was clarified by describing tetraploid sexual A. × christensenianum as a new species: *Athyrium bipinnatum* from Japan (Fig. 3).

## Materials and methods

First, the morphological characteristics of a tetraploid sexual specimen of *Athyrium bipinnatum* (*Hori 2974*) and the sexual specimens (*Hori 2975*, *2976*) described by Hori (2019) were examined. Two type specimens of *A. christensenianum*, deposited at KYO (as *Diplazium christensenianum*; Fig. 1) and TI (as *Cornopteris hakonensis*; Fig. 2), were also examined. Furthermore, based on morphological characteristics, more specimens of *A. bipinnatum* were identified at MAK and MBK, and from online images at PE herbarium (PE: http://pe.ibcas.ac.cn/en/), Taiwan Forestry Research Institute herbarium (TAIF: http://taif.tfri.gov.tw/search.php), Collection Database of Specimens and Materials (TNS: http://db.kahaku.go.jp/webmuseum/), and from those in JSTOR Global Plants (https://plants.jstor.org/) and the Global Biodiversity Information Facility (GBIF: https://www.gbif.org). Additional samples were also collected in the field and cultivated samples were taken from Koishikawa Botanical Garden of Tokyo University. For the conservation assessment, the area of occupancy (AOO) and extent of occurrence (EOO) were estimated using GeoCAT (Bachman et al. 2011), with the default settings for grid size applied. The morphology of scales and spores was observed using a KEYENCE VHX-D500.

## Taxonomic treatment

### Athyrium bipinnatum

Taxon classificationPlantaePolypodialesAthyriaceae

K.Hori sp. nov.

DE234CD5-67DC-5277-973E-050C6A269780

urn:lsid:ipni.org:names:77209707-1

Figure 4

#### Diagnosis.

*A. bipinnatum* is similar to A. × christensenianum (Koidzumi 1924, Nakai 1930, Kato 1979, Serizawa 1981) as it has serrated pinnae and exindusiate sori. However, *A. bipinnatum* has more short stipes (10–20 cm long), smaller blades (20–40 cm × 14–20 cm), 2-pinnate pinnae in the middle of the blades, and fronds with dark green adaxial surfaces. In contrast, A. × christensenianum has longer stipes (25–40 cm long), larger blades (30–60 cm × 25–40 cm), 2-pinnate pinnatifid pinnae in the middle of the blades, and fronds with light green adaxial surfaces.

#### Type.

Japan. Shikoku: Kochi prefecture, Ochi town, Mt. Yokogura, 33°32'11"N, 133°12'33"E, alt. 664 m, planted coniferous forest containing *Cryptomeria japonica* (Thunb. ex L.f.) D.Don, on soil, 29 Jun 2019, *K. Hori 3277* (holotype: MAK466762; isotype: MBK).

#### Description.

*Terrestrial, summer green fern*. *Rhizomes* creeping, occasionally 2-branched, stramineous, 8–15 × 0.8–1.5 cm, closely set with roots and persistent, densely clothed by old stipe bases, glabrous; *fronds* 1–3 per rhizome; *stipes* stramineous or slightly purple-red, 7–20 × 0.3–0.8 cm, sparsely clothed with stramineous to dark brown scales at the base (3–5 × 1–1.5 mm), lanceolate; *blades* dark green adaxially, 3-pinnatilobed at the base, 2-pinnate in the middle to upper section, pinnatifid at the apex, 20–40 × 14–20 cm, deltoid; *rachises* stramineous or slightly purple-red, dark brown, abaxially densely pilose, several projections 0.1 mm long on the adaxial surface at the bases of costae; *pinnae* 7–11 pairs, ascending, lanceolate, opposite from the base to the middle, alternate in the apex section of the blade, petioled (1–3 mm), sessile near the apex, closely spaced or overlapping, lowest pinnae slightly reduced, second lowest pair usually largest, 6–16 × 1.5–3 cm; *pinnules*, alternate on the basal and middle sections of the blade, opposite on the apex of the blade, 10–20 pairs on the basal and middle sections of the blade, 8–10 pairs on the apex of the blade, reduced distally, ovate to lanceolate, shallowly serrate to lobed, margin subcartilaginous, vein-free, close to or reaching to the margin, 3–8 pairs in the middle lobe; *the most basiscopic pinnules on the lowest pinnae* clearly short, independent from the costa, 0.6–1 × 0.3–0.5 cm; *sori* tend to appear on the abaxial surface of the middle part of blades, short linear- or oblong-shaped, single, 1.5–5 mm long, on the apex or middle of veinlets, 1–5 per ultimate segment, exindusiate, rather persistent; *spores* regular shaped, 64 regular-shaped spores per sporangium, fertile, ovoid, wrinkled, 42–54 μm × 29–35 μm, brown.

**Figure 4.**
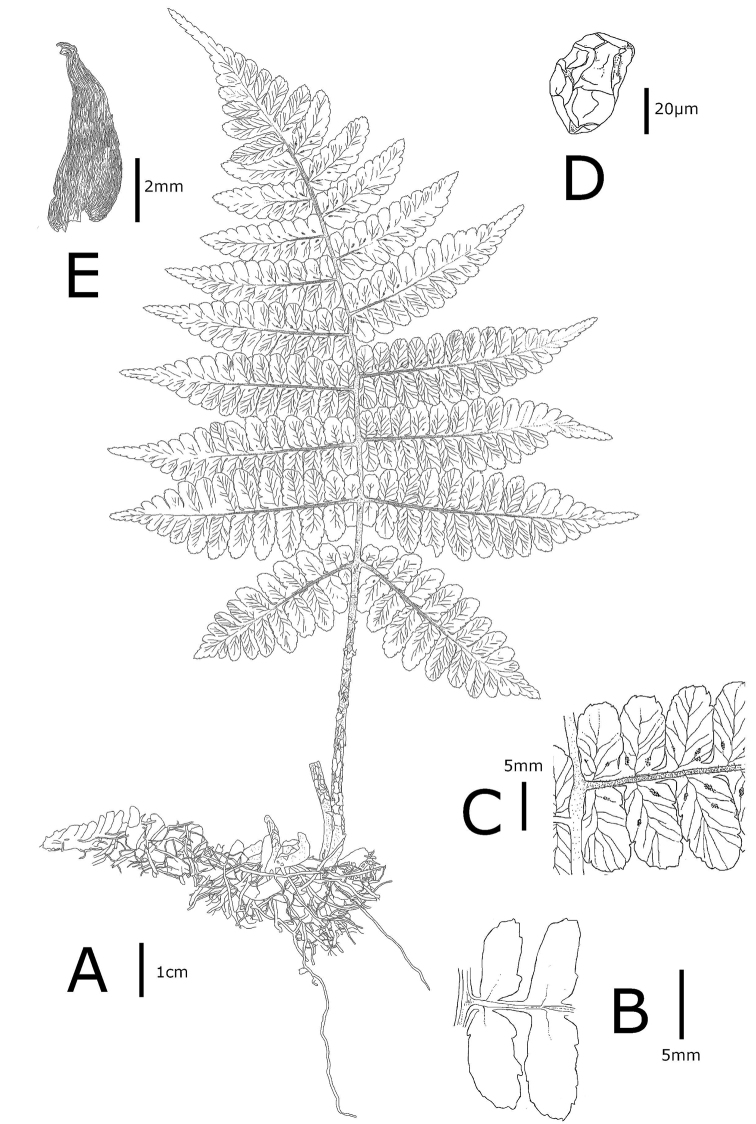
*Athyrium bipinnatum* K.Hori. **A** Habit **B** detail of adaxial pinnule **C** detail of abaxial pinnule **D** lower stipe scale **E** spore. **A–E** from the holotype (MAK466762) (illustration by K. Hori).

#### Etymology.

*Athyrium bipinnatum* is named as a new species with bipinnate blades, which distinguish it from A. × christensenianum. This new species could be one of the parents of A. × christensenianum (Hori 2019).

#### Specimens examined.

**Japan. Honshu**: Wakayama pref., Ito county, Kouya town, Mt. Kouyasann, Okunoin, alt. 800 m 7 Aug1954, coll. *M. Tagawa* (NMNH 01529356, image!); *loc. cit.*, ca. 800 m alt., 7 Aug 1954, coll. *S.K.* (MAK139!); *loc. cit.*, on soil under planted coniferous forest containing *C. japonica*, alt. 800 m, 19 Aug 2019, *K. Hori 3324* (MAK); Mie pref., Taki county, Miyagawa village, Doukuradani, alt. 1000 m, 6 Aug 1962, coll. *Y. Higuchi* (TNS471359, image!); Nara pref., Tenkawa village, Mt. Gyojagaeridake, 15 Jul 1954, coll. *Iwastuki* (MAK 26788!); *loc. cit.*, Dorokawa, Mitarai valley, on soil under planted coniferous forest containing *C. japonica*, alt. 840 m, 20 Aug 2019, *K. Hori 3326* (MAK). **Shikoku**: Kochi pref., Ochi town, Mt. Yokogurayama, 24 May 1956, *Iwatsuki 1603* (PE, NMNH, MNHN, images! MAK! TI!); *loc. cit.*, on soil under planted coniferous forest containing *C. japonica*, alt. 800 m, 30 May 2019, *K. Hori 2974*, *2975*, *2976* (MAK, MBK); *loc. cit.*, Aki county, Umaji village, Yanase, 19 June 1983, *Y. Koukami M83-163* (MBK); *loc. cit.*, Muroto city, Kiragawa town, Nishinogawa river, l4 July 1976, *Y. Kazuoki 6614* (MBK). **Kyushu**: Fukuoka pref., Maebara town, Mt. Haganeyama, cultivated at Koishikawa Botanical Garden of Tokyo University, 17 June 2019, *K. Hori 3268* (MAK); *loc. cit.*, Kaho county, Chikuho town, Mt. Toishiyama, 17 July 1966, *S. Tsutsui 2655* (TNS, image!); Oita pref., Takeda city, Kuju town, Mt. Kurodake, alt. 1140, 31 Aug 1997, *S. Tsuji TJ-4346* (MAK).

**Key to *A. bipinnatum* and closely related species in Japan**

**Table:** 

1	Blades 2-pinnate pinnatifid in the middle section, yellowish green or light green adaxially	**2**
–	Blades 2-pinnate or 1-pinnate pinnatifid in the middle section, dark green adaxially	**3**
2	Blades yellowish green adaxially, pinnule in the middle section of blade deeply serrated on margin, spores regular	***A. crenulatoserrulatum***
–	Blades light green adaxially, pinnule in the middle section of blade shallowly serrated on margin, spores abortive	**A. × christensenianum**
3	Blades 2-pinnate, pinnae straight, sori often single	***A. bipinnatum***
–	Blades 1-pinnate pinnatifid, pinnae curved to the apex, sori often lobed	***A. decurrentialatum***

#### Distribution and ecology.

**Figure 5.**
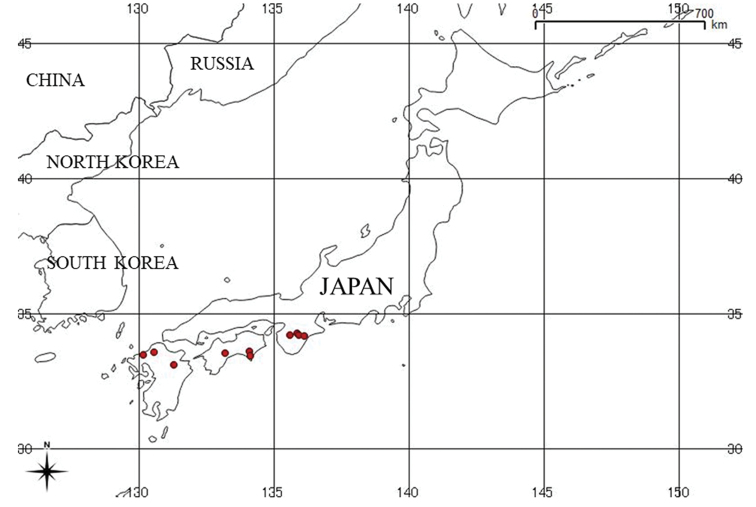
Map showing the known distribution of *Athyrium bipinnatum* in Japan.

*Athyrium bipinnatum* is known from the western part of Honshu, Shikoku, and Kyushu in Japan (Fig. 5). It was observed to grow on soil under planted coniferous forest containing *Cryptomeria japonica* at ca. 600–1100 m alt. This species is endemic to Japan.

#### Conservation status.

*IUCN Red List Category.* Based on estimates from GeoCAT, the EOO of *A. bipinnatum* was 53,068 km^2^; however, this estimate should be smaller because this EOO contains an area of sea around Japan. Wild deer are known to eat *A. bipinnatum* (pers. obs.), so individual numbers within populations are currently decreasing and population reduction is occurring now. The known AOO of *A. bipinnatum* is 44 km^2^. Based on the IUCN criteria (IUCN 2001, 2012), *A. bipinnatum* falls into the Endangered (EN) category. A formal evaluation of endangerment can be summarized by the following IUCN hierarchical alphanumeric coding system of criteria and subcriteria: EN A1abc+A2+C1+C2a(i).

## Discussion

Athyrium × christensenianum was first described by Koidzumi (1924) as *Diplazium christensenianum* Koidz., from Jeju (Quelpaert)-Island, South Korea (Fig. 1). Subsequently, Nakai (1930) described *Cornopteris hakonensis* Nakai, from Hakone, Japan (Fig. 2). I examined type material of both taxa, and found that they represent the same hybrid in having large size of stipes, blades, 2-pinnate pinnatifid pinnae in the middle of the blades. *Athyrium bipinnatum* is distinguished from them in having shorter stipes, smaller blades, and by its 2-pinnate pinnae in the middle of the blades.

**Figure 6.**
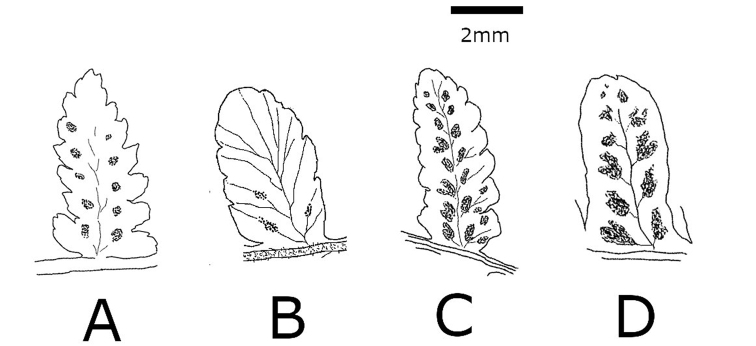
Abaxial surface of pinnule and sori of **A***Athyrium crenulatoserrulatum***B***Athyrium bipinnatum*, **C**Athyrium × christensenianum**D***Athyrium decurrentialatum* (illustration by K. Hori).

**Figure 7.**
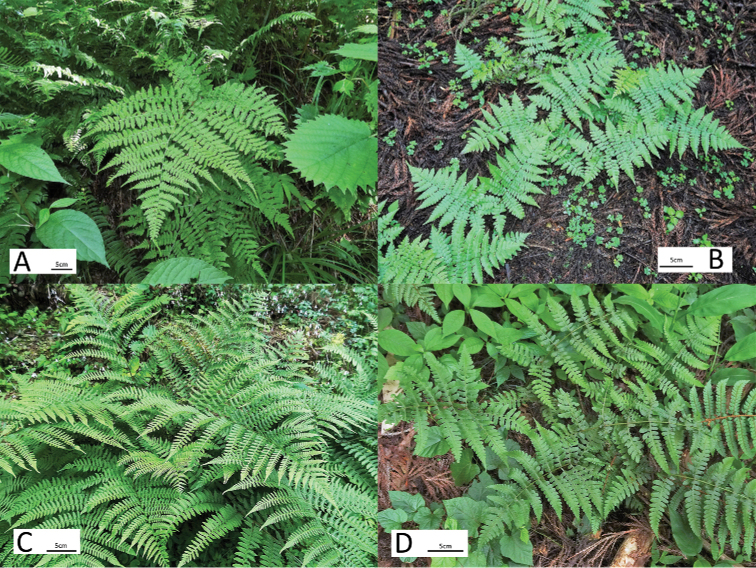
Wild plants of **A***Athyrium crenulatoserrulatum***B***Athyrium bipinnatum***C**Athyrium × christensenianum**D***Athyrium decurrentialatum*.

*Athyrium bipinnatum* is of hybrid origin between a diploid sexual *A. crenulatoserrulatum* and an extinct or undetected diploid sexual *A. decurrentialatum*, and it is one of the parents of A. × christensenianum (Hori 2019, Fig. 3). *Athyrium bipinnatum* is clearly smaller than A. × christensenianum, and its morphological characteristics, which include 2-pinnate blades, are intermediate between those of *A. crenulatoserrulatum* and *A. decurrentialatum* (Figs 6, 7, Table 1). In contrast, the morphological characteristics of A. × christensenianum, which include 2-pinnate pinnatifid blades, are intermediate between *A. bipinnatum* and *A. crenulatoserrulatum*; the large size of this plant indicates this hybrid has heterosis. *Athyrium bipinnatum* and *A. crenulatoserrulatum* each have shorter stipes (10–20 cm and 20–30 cm long, respectively) and smaller blades (20–40 cm × 14–20 cm and 30–35 cm × 25–30 cm, respectively) than A. × christensenianum (25–40 cm long stipes and 30–60 cm × 25–40 cm blades) (Table 1).

**Table 1.** Morphological comparison among *A. bipinnatum* and related species.

**Characters**	***A. bipinnatum***	***A. × christensenianum***	***A. crenulatoserrulatum***	***A. decurrentialatum***
Stipe length (cm)	7–20	25–40	20–30	20–30
Pinna size (cm)	6–16× 1.5–3	15–30 × 5–9	10–20 × 5–8	8–15 × 3–5
Pinna stalk length (cm)	0.2–0.4	0.2–0.4	0.4–0.9	0–0.2
Blade size (cm)	15–40 × 14–20	30–60 × 25–40	30–35 × 25–30	20–35 × 15–25
Blade color adaxially	dark green	light green	yellowish green	dark green
Blade division medially	2-pinnate	2-pinnate pinnatifid	2-pinnate pinnatifid	1-pinnate pinnatifid
Pinnule division medially	shallowly serrated	deeply to shallowly serrated	deeply serrated	shallowly serrated or entire
Sori	single	sometimes lobed	single	often lobed
Spore shape	regular	irregular	regular	regular
Spore size (μm)	42–54 × 29–35	30–44 × 22–39	25–37 × 16–20	27–45 × 21–35

Contrary to the findings of the present study, Park and Kato (2003) reported regular-shaped and germinative spores in their description of A. × christensenianum. Here, regular-shaped spores could not been found from dozens of A. × christensenianum herbarium specimens; however, regular-shaped spores were found from several specimens of *A. bipinnatum*, *A. crenulatoserrulatum*, and *A. decurrentialatum*. Therefore, it is possible to speculate that Park and Kato (2003) perhaps confused the materials of these different species when they examined spore shape.

In summary, the previously unclear taxonomy of A. × christensenianum was clarified in the present study by describing tetraploid sexual “A. × christensenianum” as the new species *A. bipinnatum*. Importantly, the conservation status analysis reported here suggests that *A. bipinnatum* may be endangered; thus, this study has relevance to the conservation of cornopteroid ferns.

## Supplementary Material

XML Treatment for Athyrium bipinnatum
